# Typhoid Fever

**Published:** 1894-06-23

**Authors:** 


### MEDICAL PROGRESS.

### TYPHOID FEVER.

**Treatment.**—Much discussion still centres round the various methods by which the duration and mortality of typhoid fever may be lessened. Burney Yeo[1] considers that the first object should be to counteract and antagonise the pyrogenic and other poisons secreted by micro-organisms which have been introduced into the body from without. He holds that the course of the fever can be and has been favourably modified by the administration of such antagonists to the specific poisons, and that the investigation of their nature and properties is the first need of the present time. The ordinary management and diet must be modified to assist in this end. Another paper at the Congress was by Juhel-Renoy of Paris,[2] advocating refrigerant treatment, which he considers reduces the total mortality in typhoid to 7 per cent. It is indicated in other infectious diseases which take on the typhoid character and is vastly superior in its results to other forms of treatment. This is partly due to its effects in favouring elimination of toxines through the kidneys. The same elimination has been sought by administering large quantities of water internally, so as to wash out toxines from the blood and intestinal canal. Maillart of Geneva,[3] following Debove and Lichtheim, gives five or six litres of water daily, or if the patient has eight tumblers of milk, he will get ten of water in addition. It does not weaken the heart but has a contrary effect. The fever is lower and is less fatal, the mouth becomes moist, swallowing is easy, the nervous system is calmed, and large amounts of urea are carried off. Patients seem to like the treatment in consequence. Statistics of treatment by cold baths after Brand's method show a death-rate of 6 or 7 per cent. This is based on a very large number of cases. Thus Bouchard speaks of having given 60,000 cold baths, while the rate under other methods ranges between 15 and 25 per cent. Wilson of Philadelphia reports a series[4] of 300 cases of adults treated by baths with a mortality of 6·6 per cent. He lays stress on the importance of commencing the treatment early to ensure its success. It should be noted, however, that he appears to have had an unusual number of hæmorrhages. Lindner[5] advises that acetic or hydrochloric acid should be added to the baths both for the comfort of the patient and for the safety of the nurses, the bed sprayed with vinegar, and H.C.L. administered internally on account of the destructive effects of acid on the bacilli. Some such plan is well worth consideration in view of the dreadful mortality among nurses attending upon typhoid patients. C. E. Page,[6] in contrasting the success obtained by the advocates of the cold bath system in over 19,000 cases with that reached by other methods, adds that he gives no food at all for one or more days, except plain water, with great satisfaction to all concerned. He points out that Gluzinski has shown that in acute febrile diseases the gastric juice has no digestive power, and believes that by withholding all food for the first few days until the patient is really hungry, "all curable cases would be shortened, complications lessened, and quite a proportion of cases aborted absolutely." Under this plan baths are seldom required after the first day or two, sponging or a damp bandage round the abdomen while the temperature is over 101 deg. is usually all that is needed, as the temperature rarely reaches 103 deg. after that time. He points out that as the patient will have very little thirst, if treated after this method, it is desirable to order definitely half a tumbler of hot soft water every hour. His view of the comparative value of the treatments by antipyretics, and by cold baths with primary fasting is that under antipyretics the patient is "able to die with an almost normal temperature," while the cold bath sends tone and vigour to the whole system. He, moreover, appears to accept the German theory that a fever patient cannot catch cold, but insists on the need of constantly rubbing the patient's body during the administration of a cold bath. The whole paper, though rhetorical in style, is suggestive and valuable.

In private practice there are very great difficulties in the way of cold baths. Sponging does not suffice to lower the temperature, and therefore Dr. Curnow's[7] plan of the ice pack is specially valuable. An ordinary sheet folded in four layers, rung out in ice water and packed between the layers with pounded ice, is wrapped round the body, and the patient left in it till his temperature has fallen to 102 deg. or 103 deg., but not afterwards. This packing has gone on almost constantly for eight days, with no bad results. He has almost given up antipyretics, many of them being depressant, and quinine in 20 grain doses being unreliable. In hæmorrhage from the bowels he always relies on iced compresses and opium in place of astringents. He does not allow any relaxation of diet until the temperature has been for ten days normal. He adds in turn bread, custard, eggs, fish, and meat at intervals of three days, taking some two or three weeks to return to ordinary diet. Alcohol he gives only when there are special indications, such as a weak first sound, but then he gives it very freely. Thus one patient received 388 ounces of brandy, and twenty-one pints of champagne during his illness. Treatment by free and thorough evacuation of the bowels daily by purgatives is advocated by Thistle of Toronto.[8] He reminds us that the typhoid toxine is a caustic with narcotic effects on living beings. Hence the ulceration of the bowels is prevented by removing this substance (and the germs producing it) from the intestines, the blood and lymph, and diluting as much as possible the toxine already in the tissues, and finally by destroying the remaining bacilli. He gives a short account of forty-two cases without death, hæmorrhage, or perforation, and in which the normal temperature was on the average attained on the twelfth day. All these were treated by free purgation, copious draughts of water, and generally by five or ten grains of salol every three or four hours. Various ordinary purgatives were used—magnesium sulphate, cascara, calomel, &c., and he denies that perforation or hæmorrhage is likely to be caused by purgation which removes the caustic toxine from the injured spot, relieves peristalsis and distension, and does not increase blood pressure in the vessels, whose walls have been already ulcerated. It would be interesting to know how many of his cases were children.

The practitioners who rely on drug treatment have not brought forward any very new methods. We may mention in passing the formula of Dr. A. M. Anderson, of Dundee, which is said to reduce the mortality by half, and to bring down the temperature to normal in twelve days.[9] It contains ten grains each of salol and chlorodyne and a drachm of the lac bismuthi (Symes), which are given every two hours, and the results are said to be most satisfactory. R. Bell,[10] of Glasgow, prescribes naphthaline in two four-grain palatinoids every four hours, and a tablespoonful of saturated chloroform solution every hour. He reduces the temperature by phenacetin (grains ten every four hours) when the temperature is above 101 degrees.

R. H. Quill[11] reports good results from a combination of carbolic acid and chloroform, three minims of the former with an ounce of chloroform water and ten drops of spirits of chloroform every two hours for five, seven, or ten doses daily. He gives nine grains of calomel in divided doses during the first week. The mortality in[12] the London Temperance Hospital has been 14·5 per cent. of the cases in twenty years, but Sir B. W. Richardson points out that numerous observations are needed before we can formulate any reliable conclusions as to the good or evil cf alcohol in treatment. Here we may mention Lewaschew's paper at the Congress[13] on the treatment of typhus by fuchsine. He believes that by giving one gramme of fuchsine divided into six or twelve doses during the twenty-four hours the evolution of the fever may be shortened and even aborted. He hopes that the remedy may be of equal value in septicæmia. Dr. E. N. Nason[14] discusses the value of antiseptics from the point of view of their local action, not only on the specific organisms, but also on the numerous putrefactive ones which flourish in the lesions of the intestines. Even if we cannot destroy them in the blood it is reasonable to cut off the source of their invasion by destroying their colonies in the intestines. He found that Burney Yeo's chlorinated quinine in shortening the duration, moderating the fever, and preventing complications showed a specific action on the disease of an unmistakable kind. Dr. T. E. Woodbridge[15] claims to be able to abort typhoid if seen during the first week by giving first a powder every half hour composed of minute doses of calomel, thymol, menthol, guiacol, and podophyllin, followed by a eucalyptol and guiacol draught every three hours,and if necessary by a mild diuretic mixture. There is apparently nothing in all this to prevent a patient from recovering, but the drugs given separately show no token of such efficacy as our author claims for them. One other antipyretic may be referred to. Da Costa brings forward the external use of guiacol as a substitute for cold baths. In promptness of action it is, he says, inferior to the cold bath, but the reduction of temperature is more permanent, and there is less disturbance to the patient in applying the remedy. Thirty drops is rubbed into the skin of the thigh or abdomen by a soft brush and covered with waxed paper. The skin should be first washed in soap and water, and the amount of guiacol to be used depends on the height of the temperature.

We ought in speaking of these almost unknown agents to refer to the report on the older antipyretics phenacetin, antipyrin, &c., in the *British Medical Journal*, Jan. 13. It is at least noteworthy that no accidents from their use have been reported of late, nor have we seen any such statement made against effective reliable doses of quinine of 30 or 40 grains in recent papers.

[1] Med. Week, April 6, 1894. [2] Med. Rec. April 21, 1854. [3] Med. Rec., April 21, 1894. [4] Med. Chron., March, 1894. [5] Deutsch Med. Zeit., Dec., 1893. [6] Med. Rec., April 21, 1894. [7] Clin. Jour., Mar. 7, 1894. [8] Med. Rec., Mar. 10, 1894; March 31 and April 21. [9] Chemist and Drug., April, 1894. [10] Provinc. Med. Jour., Feb. 1, 1894. [11] B. M. J., April 28, 1894. [12] Lancet, May 12, 1894. [13] Med. Week, April 13, 1894. [14] Birming. Med. Rev., Mar., 1894. [15] Clinical J., April 4, 1894. [16] Practitioner, May, 1894.

